# A Novel Physiotherapy Approach for Enhancing Mobility in a 53-Year-Old With Down Syndrome: A Case Report

**DOI:** 10.7759/cureus.56264

**Published:** 2024-03-16

**Authors:** Anushri R Patil, Snehal Samal, Nikita H Seth

**Affiliations:** 1 Neuro Physiotherapy, Ravi Nair Physiotherapy College, Datta Meghe Institute of Higher Education & Research, Wardha, IND

**Keywords:** brain gym exercises, strength-training, case report, physiotherapy, down syndrome

## Abstract

Down syndrome (DS) is a genetic condition developing from a supplementary chromosome 21, referred to as trisomy 21. It ranks among the most prevalent developmental disabilities. People with DS often live inactive lifestyles, not meeting the weekly physical activity guidelines. With age, they face increased risks of cardiovascular disease and osteoporosis, as well as neurological and orthopedic concerns. Physiotherapy is especially important for improving balance, coordination, strength, and endurance in adults over the age of 50. Our approach consisted of a three-week regimen that included strengthening exercises based on the DeLorme strength training principle, balance exercises with perturbation and treadmill training, and coordination exercises with equilibrium and non-equilibrium movements. We evaluated outcomes using measures such as the Berg Balance Scale, Timed Up and Go test, and Functional Independence Measure, which were performed before and after the physiotherapy intervention. We present a case study of a 53-year-old woman to demonstrate the importance of physiotherapy in making lifestyle changes and improving strength, balance, and endurance, thereby improving overall quality of life through tailored interventions.

## Introduction

John Langdon Down first described Down syndrome (DS) in 1866 and identified it as a trisomy of chromosome 21 in 1959. DS is a main cause of intellectual disability [1,2]. According to recent national prevalence estimates, 14.47 infants out of every 10,000 live births are diagnosed with DS. Children with DS may exhibit peculiar physical characteristics, immune and endocrine system deficiencies, and delayed cognitive development [3]. However, over the last century, their life span has significantly increased from nine years in 1929 to 60 years in 2002 [4-6]. Given this age group change, DS can no longer be viewed as a “pediatric” disease but rather as a condition that affects the entire lifetime [7]. Adults with DS are distinguished by the presence of several clinical conditions that frequently require the use of several medications and treatments, particularly psychotropic drugs, as well as a lack of adequate social and family support [5].

Physical conditions related to DS include musculoskeletal disorders, obesity, and cardiovascular disorders [8,9]. Adults with DS are more likely to develop balance impairments, problems with coordination, and musculoskeletal problems as they age. Due to this, activities of daily living are affected. Balance and strength are also the most common problems that individuals face at later stages of DS [10,11]. Physiotherapy programs seem to have the ability to improve the overall health of adults with DS, thereby increasing their quality of life. It includes multidisciplinary interventions such as strength training, aerobic training, cognitive training, treadmill training, and coordination exercises. By tailoring interventions to the individual needs of those with DS, physiotherapists not only contribute to the physical well-being of their patients but also foster improvements in overall health and social participation [12]. This case report highlights the case of a 53-year-old female with DS, emphasizing the importance of physiotherapy in tackling challenges and improving quality of life.

## Case presentation

Patient information

A 53-year-old female, known for having DS, presented to the hospital with chief complaints of difficulty in balance while walking and standing. All her gross motor and fine motor milestones were delayed in her childhood. The onset of these symptoms was gradual, and the family reported that symptoms had been progressively worsening over the last few months. According to the patient’s mother, the patient’s difficulties with balance had become more frequent, making it challenging for her to do daily activities independently. She had experienced several instances of stumbling and had required assistance while walking to prevent falls. The mother mentioned that there had been no recent incidents of trauma or injury. In addition to the balance difficulties, she reported a disturbance in the patient’s gait. She appeared unsteady and tended to lean to one side while walking. The patient did not have associated symptoms such as dizziness, vertigo, headaches, or changes in vision. There was no history of recent illness or infection. Her medical history was significant for DS, and she had been regular with routine medical checkups.

Clinical findings

The examination was conducted with the informed consent of the patient’s relative. The patient was assessed in a sitting position. Observation revealed features of DS. Growth failure (short stature), broad flat face, flat posterior aspect of the head, slanting eyes, short nose, epicanthic eye fold, dental anomalies (small and arched palate), large wrinkled tongue, special skin ridge patterns, short and broad hands, and big toes widely spread were observed. The posture of the patient showed a stooped posture at the trunk. The patient had had a known case of hypothyroidism for two years. Examining cognition, the patient was mentally retarded, as the intellectual quotient score was below 69, which is considered very low. Using the Montreal cognitive scale, the score was 2/30. On sensory examination, all sensations and reflexes were intact. Gait examination revealed a short steppage gait with reduced cadence, an absent heel strike, and an absent toe-off. Balance was assessed with the help of the Berg Balance Scale (BBS). Manual muscle testing was used to assess the bilateral upper and lower limbs. Deep tendon reflexes were assessed for the upper limb and lower limb, which were diminished. Table 1 shows the assessment findings of manual muscle testing of bilateral upper and lower limbs.

**Table 1:** Manual muscle testing for upper limb and lower limb (pre-physiotherapy intervention) Grade 2: movement through a complete range of motion for the muscle being tested; Grade 2+: holds against slight pressure in the test position; Grade 3-: gradual release from the test position

Muscles	Left side	Right side
Shoulder		
Flexors	3-	3-
Extensors	3-	3-
Abductors	2	2
Adductor	2	2
Elbow		
Flexor	3-	3-
Extensors	3-	3-
Wrist		
Flexors	2+	2+
Extensors	2+	2+
Hip		
Flexors	3-	3-
Extensors	2+	2+
Abductors	3-	3-
Adductors	3-	3-
Knee		
Flexors	2+	2+
Extensors	2+	2+
Ankle		
Plantarflexors	2+	2+
Dorsiflexors	2+	2+

Physiotherapy management

A tailored physiotherapy treatment was provided for three weeks, targeting muscle strengthening, balance training, gait training, endurance training, coordination exercises, and brain gym exercises. Since patients with DS have learning difficulties, exercises were taught with visual demonstrations, focusing on teaching one thing at a time, practicing and rehearsing, simulating multisensory feedback, generalizing training rather than task-specific training, and improving reaction time. Table 2 shows a detailed physiotherapy intervention.

**Table 2:** Physiotherapy protocol given for three weeks DS, Down syndrome; PNF, proprioceptive neuromuscular facilitation

Goals	Therapeutic exercises
Phase I: Day 1, Week 1	
Precaution	Each session should not be more than 30 minutes, avoid ballistic stretching, and avoid constant and progressive resistance exercises.
Patient education	Guidance is given to the patient’s relative about DS, its causes, and the importance of physiotherapy. The patient’s relatives are sensitized about physiotherapy and adherence to exercise programs.
To improve muscle strength	Muscle strengthening exercises with the help of half-kilogram dumbbells for upper limb and lower limb (10 repetitions × one set), lower limb closed kinetic chain exercises (10 repetitions × one set), and PNF for D1 and D2 flexion and extension patterns for upper limb and lower limb (hold-relax technique)
To improve posture	Shoulder shrugs, scapular sets, stretching exercises for the spine for lateral flexion, and rotation to the right side (10 repetitions × one set)
To improve endurance	Breathing exercises (deep breathing) (10 repetitions × one set), diaphragmatic breathing (10 repetitions × one set), thoracic expansion exercises (10 repetitions × one set), and static cycling (five minutes)
Phase II: Week 2–Week 3	
To improve balance	Perturbation training (five repetitions × one set), equilibrium exercises (10 repetitions × one set), sitting in a comfortable position, weight shifting in all directions, sitting (multidirectional functional reach), picking an object up off the floor, feet together (narrow base of support), standing with one foot directly in front of the other (tandem position), walking backward, walking sideways, marching in place, walking on toes, and stepping over or around obstacles
To improve coordination	Coordination (non-equilibrium) exercises: finger to nose, finger to finger, alternate nose to finger, finger to therapist’s finger, mass grasp, finger opposition, tapping (hand), and pronation/supination (10 repetitions × one set)
Gait training	With the help of parallel bars (five minutes), gait training on the treadmill (five minutes), and colored foot landmarks on the floor
To improve cognitive function	Brain gym exercises (10 repetitions × one set)
Home program	Breathing exercises (10 repetitions × two sets), upper and lower limb strengthening exercises (10 repetitions × one set), upper limb and lower limb ordination exercises (10 repetitions × two sets), and gait training with the help of a walker

**Figure 1:**
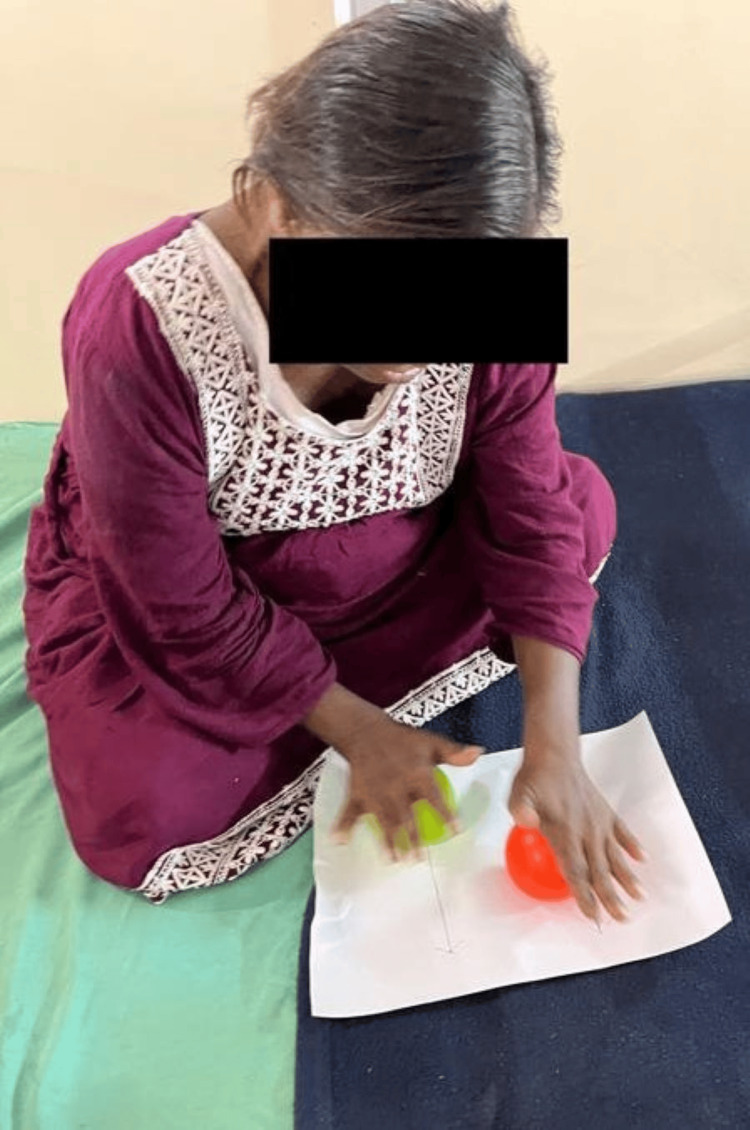
Patient performing ordination exercises using a ball

**Figure 2:**
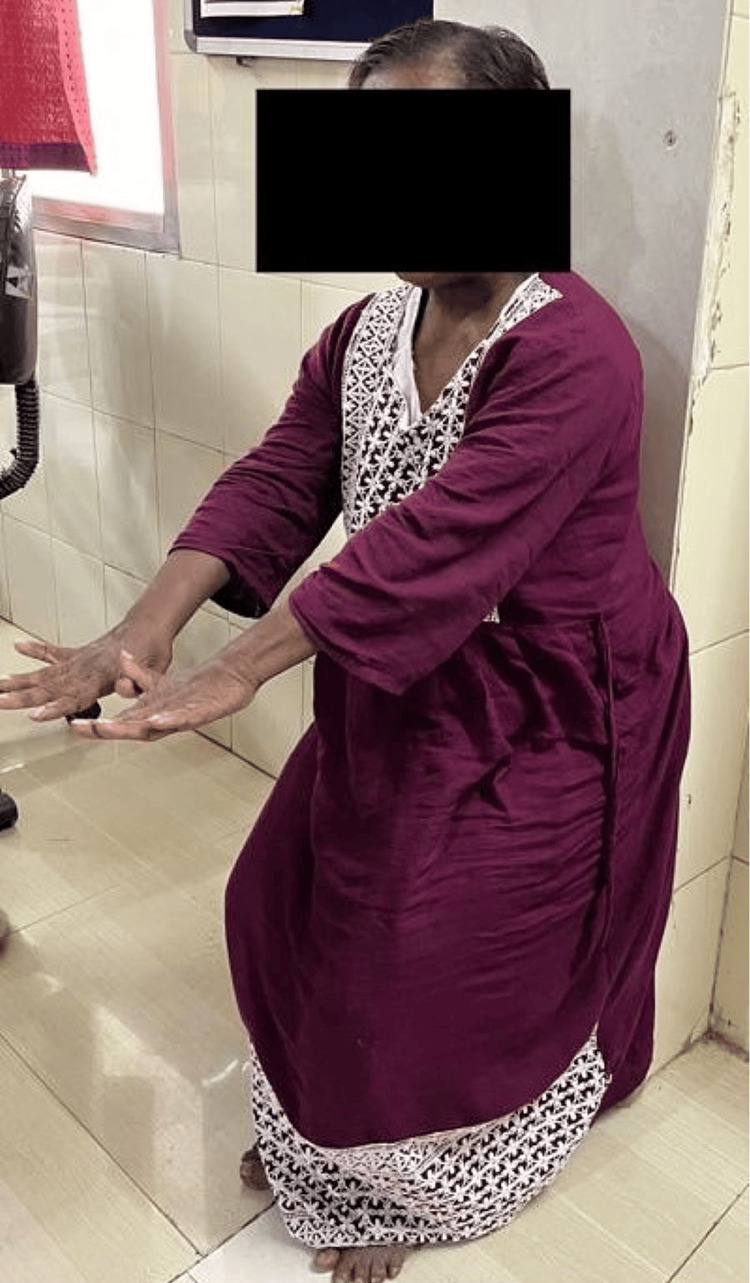
Patient performing closed kinetic chain exercises (mini squat with wall support)

**Figure 3:**
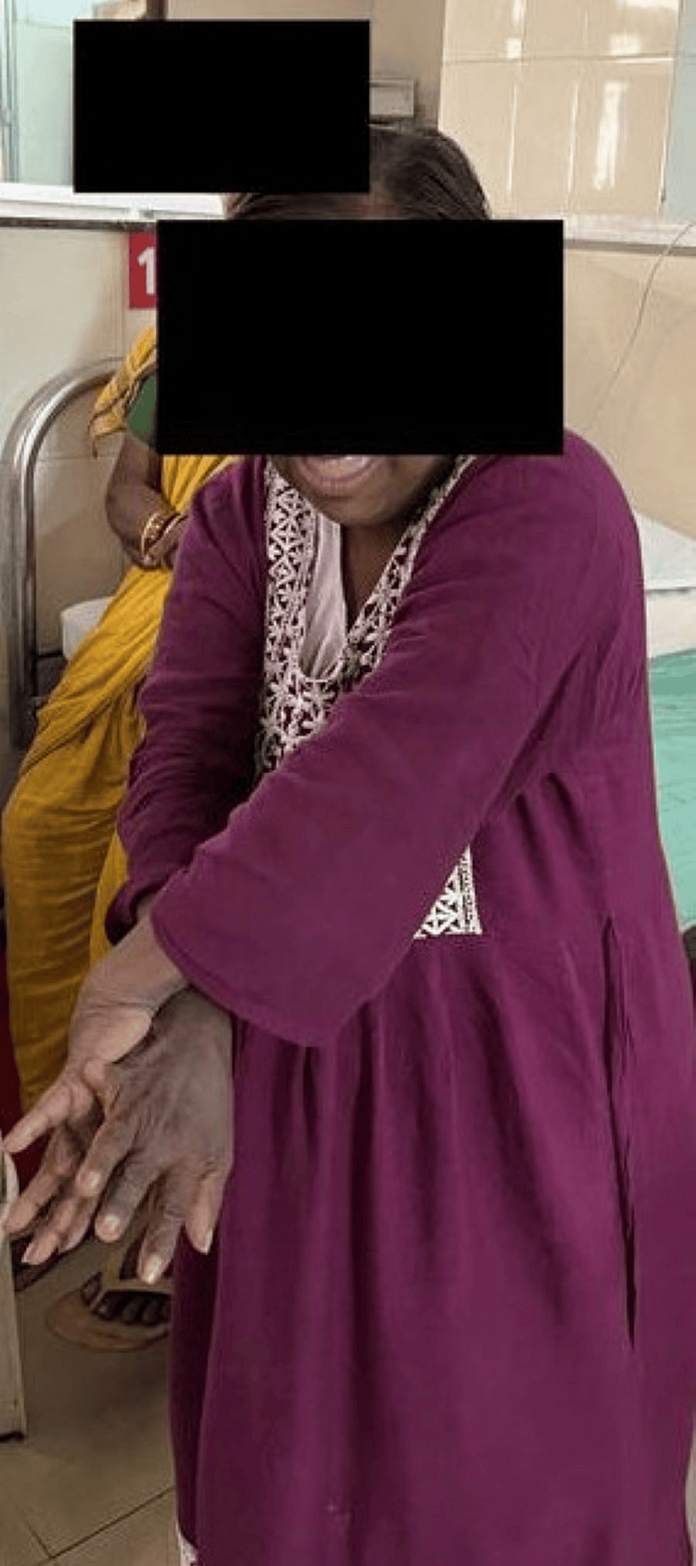
Patient performing brain gym exercise

**Figure 4:**
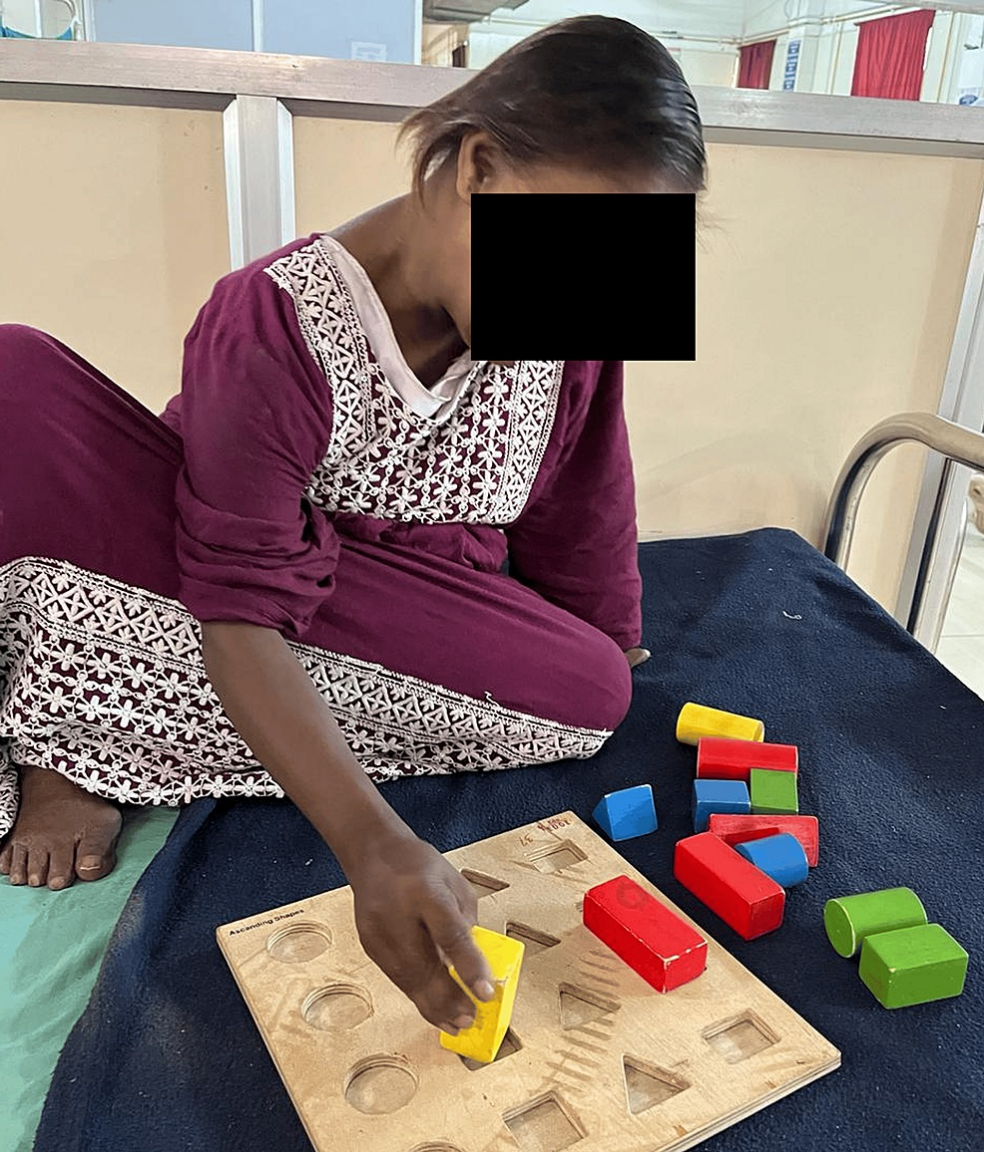
Patient performing peg board exercises for coordination

Figure 1 shows the patient performing a coordination exercise using a ball. Figure 2 highlights the patient performing a closed kinetic chain exercise. Figure 3 shows the patient performing brain gym exercises. Figure 4 shows a patient performing a coordination exercise with a peg board.

Follow-up and outcome measures

The BBS was used to assess balance. The following components of the BBS were affected: standing on one foot with eyes closed, placing the opposite foot on the stool, standing with feet together, and reaching forward with an outstretched arm. Table 3 represents outcome measures assessed before starting a physiotherapy intervention and after a physiotherapy intervention.

**Table 3:** Outcome measures BBS, Berg Balance Scale; DS, Down syndrome; FIM, Functional Independence Measure; TUG, Timed Up and Go test

Outcome measure	Pre-physiotherapy intervention	Post-physiotherapy intervention (Week 3)
BBS	44	50
FIM	70	98
TUG	15 seconds	9 seconds

## Discussion

People with DS live an average of nearly 60 years, up from 12 years in 1949. Because of the longer life expectancy, an increase in the number of adults with DS is experiencing premature age-related health problems. As a result, this aging population requires specialized healthcare [10,13]. In this case, a 53-year-old male with a diagnosed case of DS mainly focuses on balance training and gait training. As the patient faces great difficulty performing activities of daily living, it is important to gain independence and balance. We have incorporated various equilibrium exercises for balance training, progressing it to a hard task. A review conducted by Shields highlighted the multidisciplinary approach used for DS, including gait training and balance training by treadmill, aerobic exercises, progressive resistance training, and task-specific training, and found that physiotherapy was proven effective in improving the scores of the BBS and Timed Up and Go test [14,15]. Ptomey et al. conducted a 12-week study to improve cognitive function in adult patients with DS by combining aerobic exercises like jogging with music and walking, dancing, and strength exercises. They discovered improved memory and cognitive function [16].

Stander et al. studied the effect of virtual reality, combined with physiotherapy, on improving motor functions in individuals with DS. It was found to be beneficial and effective in improving the motor functions of the patients [17]. Promoting physical activity is an important part of managing quality of life. Physical activity has been shown to enhance the fitness, work capacity, and quality of life of people with intellectual disabilities [18]. Alsakhawi and Elshafey found improvement in BBS scores with the help of treadmill training and core stability exercises [19]. Sugimoto et al. incorporated neuromuscular training to investigate its effect on maximal strength, general strength, and functional mobility-related tasks in children and young adults with DS, and it showed significant improvement in muscular strength and functional ability [20]. Physiotherapy is critical to enabling people with DS to reach their full physical potential and live fulfilling lives by conducting thorough assessments and developing tailored treatment plans. In this case report, emphasizing the efficacy of physiotherapy interventions and their impact on the individual’s progress can provide valuable insights into best practices and help to advance care in this population.

Several factors contribute to limitations in the physiotherapy management of adults with DS, despite the use of various interventions such as strength training, balance training, endurance training, and gait training. One significant challenge is that many patients and their families are unaware of the benefits and importance of physiotherapy. This lack of awareness frequently leads to irregular attendance, incomplete participation, or even reluctance to engage in prescribed exercises, reducing the effectiveness of treatment. Furthermore, cognitive impairments commonly associated with DS present another challenge, as patients may struggle to complete exercises independently. As a result, there is an urgent need for tailored treatment strategies and increased education to close this gap and improve physiotherapy outcomes for people with DS.

## Conclusions

This case report highlights the significance of physiotherapy in improving the quality of life for people with DS, particularly adult females. Notably, the tailored physiotherapy regimen resulted in significant improvements in muscle tone, mobility, and overall functional abilities, improving the scores and promoting greater independence in daily activities. It is necessary to acknowledge the limitations of this case report and the significance of conducting more comprehensive research in this field. Larger sample sizes and longer intervals for follow-up are needed for future research in order to confirm that physiotherapy interventions are beneficial for people with DS in a range of age groups and functional impairment levels.
